# Long-term outcomes of endoscopic transpapillary gallbladder drainage using a novel spiral plastic stent in acute calculus cholecystitis

**DOI:** 10.1186/s12876-022-02610-5

**Published:** 2022-12-23

**Authors:** Junya Sato, Kazunari Nakahara, Yosuke Michikawa, Keigo Suetani, Yosuke Igarashi, Akihiro Sekine, Yusuke Satta, Shinjiro Kobayashi, Takehito Otsubo, Keisuke Tateishi

**Affiliations:** 1 Department of Gastroenterology, St. Marianna University School of Medicine, 2-16-1 Sugao, Miyamae-ku, Kawasaki, Kanagawa Japan; 2 Department of Gastroenterological and General Surgery, St. Marianna University School of Medicine, Kawasaki, Kanagawa Japan

**Keywords:** Acute cholecystitis, ERCP, Gallbladder, Stents

## Abstract

**Background:**

Endoscopic transpapillary gallbladder stenting (EGBS) is considered for patients with contraindications to early surgery for acute calculus cholecystitis. However, evidence regarding the long-term outcomes of EGBS is insufficient to date. The aim of the study was to evaluate the feasibility of EGBS as a bridge to or alternative to surgery when there are contraindications.

**Methods:**

We reviewed the cases of patients who underwent EGBS using a novel spiral-shaped plastic stent for acute calculus cholecystitis between January 2011 and December 2019. We retrospectively evaluated the long-term outcomes of EGBS using a novel spiral-shaped plastic stent.

**Results:**

Forty-nine patients were included. The clinical success rate of EGBS was 97%. After EGBS, 25 patients (surgery group) underwent elective cholecystectomy and 24 patients did not (follow-up group). In the surgery group, the median period from EGBS to surgery was 93 days. There was a single late adverse event with cholecystitis recurrence. In the follow-up group, the median follow-up period was 236 days. Late adverse events were observed in eight patients, including recurrence of cholecystitis (four patients), duodenal penetration by the distal stent end (two patients), and distal stent migration (two patient). In the follow-up group, the time to recurrence of biliary obstruction was 527 days.

**Conclusions:**

EGBS with a novel spiral-shaped plastic stent is safe and effective for long-term acute calculus cholecystitis. There is a possibility of EGBS to be a bridge to surgery and a surgical alternative for acute calculus cholecystitis in patients with contraindications to early cholecystectomy.

## Introduction

Acute cholecystitis is a common disease [1]. The most frequent cause of acute cholecystitis is gall stones [2]. Several treatment options are considered for acute calculus cholecystitis. The standard therapy for acute calculus cholecystitis is early laparoscopic cholecystectomy [3]. However, the number of patients who cannot undergo surgery due to high risk factors such as older age, ongoing antithrombotic therapy, and comorbidity is increasing. Therefore, gallbladder drainage is an important treatment option for the management of acute calculus cholecystitis. The standard method for gallbladder drainage is PTGBD. Although PTGBD is effective, it has not a few complications and has several problems may not be suitable for long-term treatment owing to external drainage.

Endoscopic transpapillary gallbladder stenting (EGBS) and endoscopic ultrasound-guided gallbladder drainage (EUS-GBD) are alternative therapies for patients with acute cholecystitis that do not involve an external fistula [4]. Although EUS-GBD reduced adverse events, re-intervention, and recurrent cholecystitis compared to PTGBD, EUS-GBD is still not universal [5]. On the other hands, EGBS is the procedure included in endoscopic retrograde cholangiopancreatography (ERCP), which is performed all over the world. Several reports have demonstrated the short-term efficacy and safety of EGBS [6–10]. However, the following problems need to be clarified in EGBS for acute cholecystitis: (1) the optimal stent, (2) the efficacy and safety as a bridge to surgery, and (3) the long-term outcomes for inoperable patients.

We developed a novel spiral-shaped plastic stent (SPS) for EGBS and previously reported satisfactory short-term outcomes [11]. However, long-term outcomes of SPS remain unclear.

In the present study, we retrospectively evaluated the long-term outcomes of EGBS using the novel SPS to clarify the feasibility of EGBS as a bridge to surgery or as an alternative treatment for inoperable patients.

## Patients/material and methods

### Patients

We retrospectively reviewed and included consecutive patients who underwent successful EGBS using the novel SPS for acute calculus cholecystitis between January 2011 and December 2019 at the Department of Gastroenterology and Hepatology of St. Marianna University School of Medicine. We excluded patients according to the following criteria: (1) acalculus cholecystitis, (2) use of a non-spiral-shaped stent, and (3) short follow-up period of < 30 days. All patients provided written informed consent for the procedure. This study was approved by the Institutional Review Board of the St. Marianna University School of Medicine (Approval Number: 4382).

### Indications of EGBS for cholecystitis

The first line treatment for mild to moderate acute calculus cholecystitis was early cholecystectomy. In the patients with severe cholecystitis, we performed EGBS as gallbladder drainage. In the patients with mild to moderate cholecystitis, we performed EGBS when they had one of the following conditions: (1) contraindications for early cholecystectomy, (2) severe comorbidities, (3) severe symptoms, and (4) plan to perform ERCP for acute cholangitis with choledocholithiasis.

### EGBS procedure

**Fig. 1:**
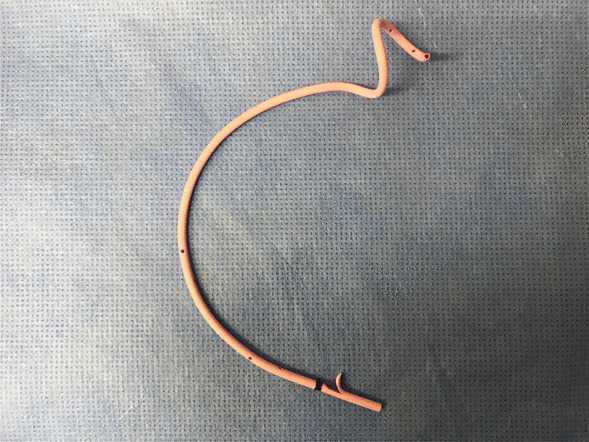
A novel spiral-shaped plastic stent (SPS)

All EGBSs were performed under the supervision of an experienced ERCP expert (more than 1000 ERCP procedures). We used a duodenoscope (JF-260V, TJF-260V, or TJF-Q290V; Olympus Medical Systems, Tokyo, Japan) and performed bile duct cannulation using conventional contrast or wire-guided cannulation. After successful biliary cannulation, cholangiography was performed to assess the shape of the common bile duct (CBD) and to determine whether the cystic duct filled with contrast. We conducted a hydrophilic guidewire (e.g., Radiforcus, Terumo Co. Ltd., Tokyo, Japan, or NaviPro, Boston Scientific, Natick, MA) through the cystic duct. Once the guidewire was inserted into the gallbladder, it was replaced with a stiff type. We then inserted a 7-French (Fr)-tapered catheter with side holes (MultiFunction Catheter, Gadelius Medical, Tokyo, Japan) over the guidewire into the gall bladder to suction the infected bile. Finally, we placed the SPS (GBest-N stent; Hanaco Medical Co., Saitama, Japan) in the gallbladder fundus (Fig. 1) [11]. The top of the novel stent had a three-dimensional spiral-shape, conceived to prevent migration. The distal end of the SPS was straight, with a flap. The semicircular shaft of the stent measured 7 Fr. There were side holes within the spiral tip and shaft of the stent. SPS lengths varied, with 11, 13, 15, 17, and 19 cm. We did not perform any scheduled stent exchanges for patients after EGBS. The patients performed EGBS were followed every 6 months unless the patients refused follow-up. Elective cholecystectomy after EGBS was performed to the patients who had indication of surgery. During the cholecystectomy, the stent was removed by the following steps: (1) Dissection of the gallbladder is started from the fundus of the gallbladder and proceeds to the neck, and only the cystic artery and duct are left. (2) The gallbladder artery is ligated with a clip and dissected. (3) Assistant inserts endoscopy to duodenum, and under observation from both the abdominal cavity and the duodenum, the stent is removed by grasping forceps through the endoscope. (4) After removal of the stent, the cystic duct is ligated and separated with a clip or end loop, and the gallbladder is removed.

### Measurements

We retrospectively assessed the patients’ backgrounds and EGBS treatment outcomes, including clinical success rates and adverse events. The diagnosis and severity of acute cholecystitis were assessed according to the Tokyo Guidelines 2018 (TG18) [12]. We defined the clinical success of EGBS as improvement (or tendency for improvement) in clinical symptoms and laboratory data within 3 days. Adverse events were divided into EGBS-related sequelae such as cystic duct injury or peritonitis and ERCP-related events. Cystic duct injury was defined as the dislocation of devices, such as guidewire or cannula, or the leakage of contrast media into the peritoneal cavity from the cystic duct lumen confirmed by fluoroscopic imaging [13]. ERCP-related adverse events occurred within 7 days of ERCP. The diagnosis and severity of ERCP-related adverse events included bleeding, perforation, pancreatitis, and cholangitis based on the consensus guidelines of Cotton et al. [14]. We evaluated the long-term outcomes of EGBS, including late adverse events and time to recurrent biliary obstruction (TRBO). Recurrent biliary obstruction defined as patients who were needed exchange or removal of EGBS because of late adverse event. Late adverse events were defined as recurrent cholecystitis, cholangitis, duodenal stent penetration, and migration. We evaluated patients with and without cholecystectomy separately. In this study, TRBO in patients without cholecystectomy included not only stent obstruction but also stent migration requiring endoscopic intervention. Furthermore, we assessed the pre-operative outcomes of EGBS as a bridge to surgery in patients who eventually underwent cholecystectomy. In addition, we also assessed the outcome of cholecystectomy after EGBS.

### Statistical analysis

Categorical variables were compared using Fisher’s exact test. Continuous variables are presented as median (range) and compared using the Mann–Whitney U-test. TRBO was calculated using the Kaplan–Meier method. Statistical significance was set at *P* < 0.05. Statistical analyses were performed using R version 3.4.1 software (R Foundation, Vienna, Austria).

## Results

**Fig. 2:**
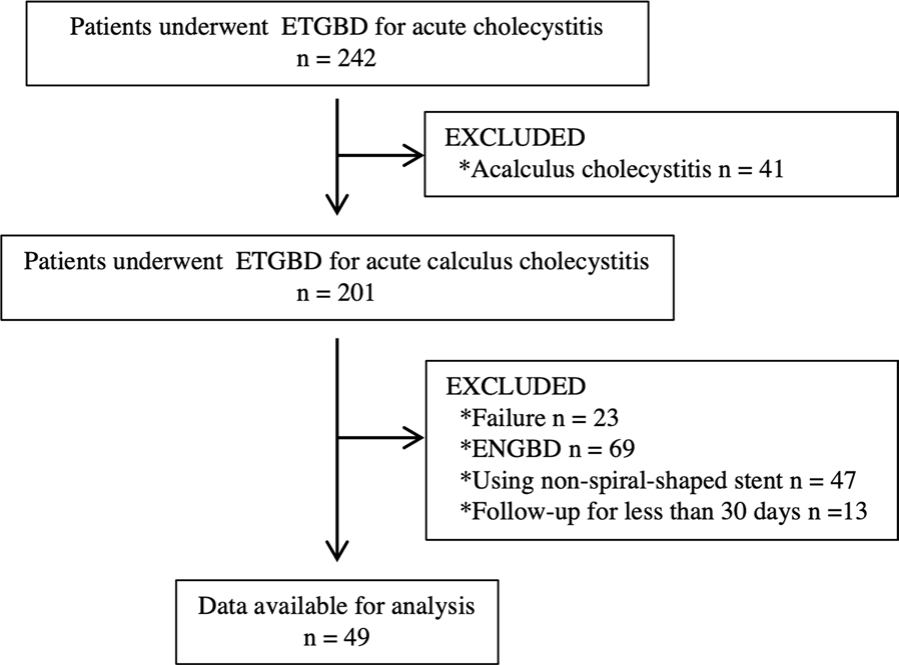
Patient flowchart. ETGBD, endoscopic transpapillary gall bladder drainage; EGBS, endoscopic gallbladder stenting; ENGBD, endoscopic nasobiliary gallbladder drainage

Between January 2011 and December 2019, 242 patients underwent endoscopic transpapillary drainage. Among them, 193 patients were excluded from the study for the following reasons: (1) acalculous cholecystitis (41 patients), (2) Technical failure of ETGBD (23 patients), (3) endoscopic nasobiliary drainage (69 patients), (4) use of a non-spiral-shaped stent (47 patients), and (5) follow-up for < 30 days (13 patients). Thus, 49 patients were enrolled in this study (Fig. 2). The baseline patient characteristics are shown in Table 1. Mild cholecystitis was diagnosed in 12 (24%), moderate in 31 (63%), and severe in 6 (12%) patients. The stones were found in the cystic duct in five patients (10%) and the gall neck in nine (16%). CBD stones were present in 17 (35%) patients. EGBS was performed instead of cholecystectomy because of CBD stones in 16 patients, antithrombotic therapy or coagulopathy in 16 patients, severe comorbidity in 10 patients, poor performance status in five patients, refused surgery by one patient, and suspected malignant gallbladder lesion in one patient.

**Table 1:** Baseline characteristics

Characteristic	All patients n = 49	Patients without cholecystectomy after EGBS n = 24	Patients with cholecystectomy after EGBS n = 25
Male: Female	27: 22	13: 11	14: 11
Median age, years (range)	75 (46–91)	78 (46–91)	73 (48–85)
PS (ECOG) 0–2: 3–4	38: 11	14: 10	24: 1
Antithrombotic therapy	23 (47%)	13 (54%)	10 (40%)
Severity of cholecystitis			
Mild	12 (24%)	11 (46%)	1 (4%)
Moderate	31 (63%)	9 (38%)	22 (88%)
Severe	6 (12%)	4 (17%)	2 (8%)
Impaction of the gallstone			
In cystic duct	5 (10%)	4 (17%)	1 (4%)
In gall neck	9 (16%)	1 (4%)	8 (32%)
CBD stone	17 (35%)	10 (42%)	7 (28%)
Reason for EGBS			
CBD stone	16 (33%)	4 (17%)	12 (48%)
Antithrombotic therapy/coagulopathy	16 (33%)	6 (25%)	10 (40%)
Severe comorbidities	10 (20%)	4 (17%)	6 (24%)
Poor PS	5 (10%)	4 (17%)	1 (4%)
Other	2 (4%)	0	2 (8%)

*PS* performance status, *ECOG* eastern cooperative oncology group, *CBD* common bile duct

The clinical success rate for EGBS was 97% (Table 2). Early adverse events were cystic duct injury in three patients (6%) and mild pancreatitis in two patients (4%). All early adverse events improved conservatively. No other events including bleeding, perforation, or cholangitis were observed.

**Table 2:** Treatment outcomes and early adverse events of EGBS

Outcomes	n = 49
Clinical success	46 (97%)
EGBS-related adverse events	
Cystic duct injury	3 (6%)
Bile peritonitis	0 (0%)
ERCP-related adverse events	
Bleeding	0 (0%)
Perforation	0 (0%)
Pancreatitis	2 (4%)
Cholangitis	0 (0%)
Severity of ERCP-related adverse events	
Mild	2 (100%)
Moderate	0 (0%)
Severe	0 (0%)

*EGBS* endoscopic gallbladder stenting, *ERCP* endoscopic retrograde cholangiopancreatography

In this study, 25 patients underwent elective cholecystectomy after EGBS (surgery group) and 24 underwent EGBS alone without cholecystectomy (follow-up group). In the surgery group, the median period from EGBS to cholecystectomy was 93 days (Table 3). Late adverse events were observed in only one patient (4%) with cholecystitis (due to stent obstruction). After EGBS, laparoscopic and open cholecystectomy were performed in 19 and 6 patients, respectively. The median operating time was 145 min. The median intra-operative blood loss was 10 ml.

**Table 3:** Long-term outcomes of EGBS in patient without cholecystectomy

Outcomes	n = 24
Median follow-up periods, days (range)	236 (48–1018)
Late adverse event	8 (33%)
Recurrent cholecystitis	4 (17%)
Cholangitis	0 (0%)
Duodenal penetration by the stent	2 (8%)
Migration	2 (8%)
Cause of stent dysfunction	
Obstruction	3
Migration	1
TRBO, days (range)	527 (322–NA)

*EGBS* endoscopic gallbladder stenting, *TRBO* time to biliary obstruction, *NA* not available

**Fig. 3:**
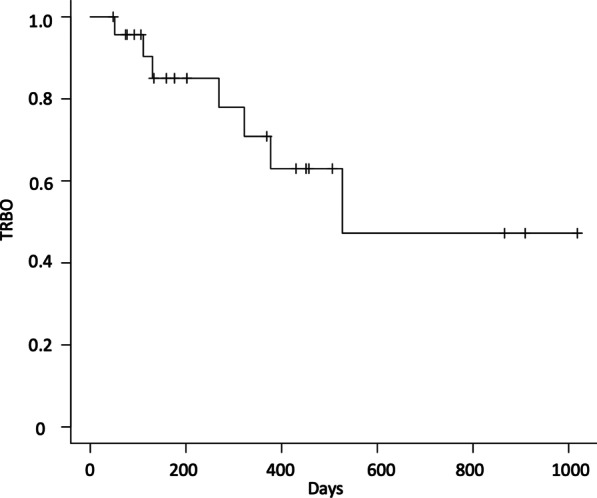
Kaplan–Meier curves of time to recurrent biliary obstruction of EGBS. EGBS, endoscopic gallbladder stenting; TRBO, time to biliary obstruction

In the follow-up group, the median follow-up period was 236 days (Table 4). Late adverse events were observed in eight patients (33%), comprising four cases of recurrent cholecystitis, two cases of duodenal stent penetration, and two case of migration. The cause of recurrent cholecystitis with stent dysfunction was obstruction in three patients and migration in one. The stent migrated toward gallbladder side in one, and toward duodenum in another. TRBO was 527 days in the follow-up group (Fig. 3).

**Table 4:** Pre-operative outcomes of EGBS in patients undergoing cholecystectomy

Outcomes	n = 25
Median period from EGBS to surgery, days (range)	93 (36–244)
Late adverse event	
Cholecystitis	1 (4%)
Cholangitis	0 (0%)
Duodenal perforation	0 (0%)
Cause of stent dysfunction	
Obstruction	1 (4%)
Migration	0 (0%)

*EGBS* endoscopic gallbladder stenting

## Discussion

In this study, we reviewed the cases of 49 patients who underwent EGBS using the novel SPS for acute calculus cholecystitis to evaluate long-term results. There are few reports available that assess long-term outcomes after EGBS for acute cholecystitis. Conway et al., Schlenker et al., and Lee et al. reported the long-term outcomes of EGBS in patients who are poor surgical candidates [15–17]. Although these studies showed the long-term efficacy and safety of the EGBS procedure, the number of patients studied was fewer than 30, which may not be sufficient for accurate analysis. In contrast, Mutignani et al. and Inoue et al. reported the long-term outcomes of EGBS in more than 30 patients. These studies have the following limitations: (1) they included both calculous and acalculous cholecystitis and (2) they used different types of stents [18, 19]. Maekawa et al. and Kim et al. reported the long-term effectiveness of EGBS using a single type of 7Fr double-pigtail biliary stent for calculous cholecystitis in 31 patients and 83 patients, respectively [20, 21]. However, all previous studies have evaluated the long-term outcomes of EGBS with biliary stents. To the best of our knowledge, ours is the first study to evaluate the long-term outcomes of EGBS for calculous cholecystitis using an SPS.

The novel SPS has several peculiarities compare with traditional stents such as double-pigtail stents and straight stents. The SPS had side holes within the spiral tip and shaft of the stent to improve drainage both gallbladder and CBD. Additionally, the three-dimensional spiral-shaped top and the straight distal end of the SPS might be effective to prevent migration. In our study, the clinical success rate of EGBS with the SPS was 97%. In a prior study, the clinical success rate of EGBS using a double-pigtail stent was 83% [18]. Stent migration rate was 4% in the current study and 9% in a previous study using a double-pigtail stent [17]. These results suggest that the SPS might offer superior outcomes than the double-pigtail stent for EGBS.

On the other hand, the late adverse event of duodenal penetration by the stent was observed in our study. This has never been reported in another EGBS paper [15–21]. Duodenal stent penetration occurred on days 269 and 527 post-EGBS. The reason for penetration could have been the long placement of the stent with a typical straight tip on the duodenal side. Fortunately, the patients with penetration were asymptomatic and required only stent exchange and preventive endoscopic clipping. An EGBS stent comparison study assessing clinical success, adverse events, and long-term outcomes is warranted to determine the most appropriate stent shape.

The outcomes of pre-operative EGBS have not previously been evaluated. In our study, the median period between EGBS and surgery was 93 days. Only one patient needed re-intervention due to stent obstruction. A previous report revealed similar early adverse events occurring in EGBS and PTGBD [22]. Although further study is needed, we think that EGBS could serve as a bridge to surgery in patients who cannot undergo early cholecystectomy, especially because of stent patency and low adverse event rate of EGBS.

We evaluated the long-term outcomes of EGBS in patients who did not undergo cholecystectomy due to poor PS or severe comorbidity. The previous reports showed late adverse event rate in patients who had no indication of surgery as 25–28% [23, 24]. As follow-up periods of our study were longer than previous reports, the late adverse event rate became slight high. In our study, the TRBO of EGBS was 527 days. A previous report showed superiority of EGBS over PTGBD for acute cholecystitis, with similar clinical success and low late adverse event rates [25]. Our data support the low late adverse event rate associated with EGBS. Although cholecystectomy is the best treatment for calculus cholecystitis, we believe that EGBS is feasible and has potential as an alternative therapy for acute cholecystitis in patients with contraindications for surgery.

Our study had several limitations. First, this was a nonrandomized, retrospective study conducted at a single center. Second, there were biases, such as those related to the treatment strategy and management of acute cholecystitis. Although we followed a continuous strategy for indication of EGBS, we could not eliminate the selection bias in this retrospective study. Third, the confounding effects of EGBS and antibiotic treatment on clinical outcomes were not evaluated in this study. Forth, each number of patients evaluated for follow-up group and surgery group may be small. Fifth, the median follow-up period of the patients in follow-up group was less than 1 year because they had difficulty to come continually to our hospital because they had problems such as poor PS or severe comorbidities. Finally, all EGBS procedures were performed by a specialist experienced in ERCP and EGBS. Therefore, the results are not likely to be universal.

In conclusion, EGBS using a novel SPS is safe and effective in the long-term for treatment of acute calculus cholecystitis. There is a possibility of EGBS to be a bridge to surgery and a surgical alternative for acute calculus cholecystitis in patients for whom early cholecystectomy is contraindicated.

## Data Availability

The data supporting the findings of this study are available from the corresponding author upon reasonable request.

## Abbreviations

EGBS: Endoscopic transpapillary gallbladder stenting
PTGBD: Percutaneous transhepatic gallbladder drainage
ERCP: Endoscopic retrograde cholangiopancreatography
SPS: Spiral-shaped plastic stent
TRBO: Time to recurrent biliary obstruction
CBD: Common bile duct
